# Pure seminoma: A review and update

**DOI:** 10.1186/1748-717X-6-90

**Published:** 2011-08-08

**Authors:** Noureddine Boujelbene, Adrien Cosinschi, Nadia Boujelbene, Kaouthar Khanfir, Shushila Bhagwati, Eveleyn Herrmann, Rene-Olivier Mirimanoff, Mahmut Ozsahin, Abderrahim Zouhair

**Affiliations:** 1 Department of Radiation Oncology, Centre Hospitalier Universitaire Vaudois (CHUV), Bugnon 46, CH-1011 Lausanne, Switzerland; 2 Department of Radiation Oncology, Centre Hospitalier Universitaire Habib Bourguiba, 3000 Sfax, Tunisia; 3 Department of Pathology, Institut Gustave-Roussy, 94805 Villejuif, France; 4 Department of Radiation Oncology, Hôpital de Sion-CHCVs, CH-1950 Sion, Switzerland; 5 Department of Pathology, Hôpital HabibThameur, 1089 Tunis, Tunisia

**Keywords:** Seminoma, treatment, radiotherapy, chemotherapy, surveillance

## Abstract

Pure seminoma is a rare pathology of the young adult, often discovered in the early stages. Its prognosis is generally excellent and many therapeutic options are available, especially in stage I tumors. High cure rates can be achieved in several ways: standard treatment with radiotherapy is challenged by surveillance and chemotherapy. Toxicity issues and the patients' preferences should be considered when management decisions are made. This paper describes firstly the management of primary seminoma and its nodal involvement and, secondly, the various therapeutic options according to stage.

## 

Testicular cancers, 95% of which are germ-cell tumors (GCT), are the most common solid malignancies affecting males between the ages of 15 and 35 years, although it accounts for only about 1% of all cancers in men [1]. In 2010 it caused an estimated 350 deaths with 8480 new cases diagnosed in the United States alone [1]. In Switzerland, and particularly in the Vaud canton, its prevalence is one of the world's highest, and is still increasing [2]. Nevertheless its origin remains poorly understood, although some environmental or genetic risk factors are suspected [3]. It is also known to be bilateral in 3% of cases [4]. GCT may consist of one predominant histologic pattern or represent a mixture of multiple histologic types. For treatment purposes, two broad categories are recognized: pure seminoma (no nonseminomatous elements present), and all others, which together are termed nonseminomatous germ-cell tumors (NSGCT). Seminoma, 80% of which are diagnosed at stage I (Table 1), is highly sensitive to both radiotherapy (RT) and chemotherapy (CHT) and, therefore, unlike many malignant neoplasms, cure is an expected outcome in the majority of cases, even with metastatic disease at presentation [3]. Its prognosis is generally good, but the treatment-induced morbidity must not be underestimated.

**Table 1:** Classification of seminomas according to UICC/AJCC and IGCCCG [7,61]

Clinical Stage	TNM (UICC/AJCC) Category					Blood tumor markers (S)		
	**T**		**N**	**M**	**S**	**LDH**	**βHCG** **(mIU/ml)**	**AFP** **(ng/ml)**

**0**	pTis	carcinoma in situ	N0	M0	-	-	-	-

**IA**	pT1	Limited to the testis and/or epididym, without lymphatic or vascular invasion, the tumor can infiltrate the tunica albuginea but not the tunical vaginalis	N0	M0	Any S level	Any LDH level	Any βHCG level	Norm.

**IB**	pT2	Limited to the testis and/or epididym, without lymphatic or vascular invasion, or spread through the tunica albuginea and invasion of the tunica vaginalis	N0	M0	Any S level	Any LDH level	Any βHCG level	Norm.
							
	pT3	Infiltration of the spermatic cord						
							
	pT4	Infiltration of the scrotal wall						

**IIA**	Any T stage		N1 (≤ 2 cm)	M0	Any S level	Any LDH level	Any βHCG level	Norm.

**IIB**	Any T stage		N1 (> 2 - 5 cm)	M0	Any S level	Any LDH level	Any βHCG level	Norm.

**IIC**	Any T stage		N1 (> 5 cm)	M0	Any S level	Any LDH level	Any βHCG level	Norm.

**IIIA/B/C**	Any T stage		Any N stage	M1a (non-regional nodes or lung metastasis)	Any S level	Any LDH level	Any βHCG level	Norm.

**IIIC**	Any T stage		Any N stage	M1b (other metastasis sites)	Any S level	Any LDH level	Any βHCG level	Norm.

**IIIC**	Mediastinal primary tumor		Any N stage	Any M stage	Any S level	Any LDH level	Any βHCG level	Norm.

LDH: lactate deshydrogenase, βHCG: Beta Human chorionic gonadotrophin, AFP: alpha-fetoprotein, T: tumor, N: nodes, M: metastasis, S:blood marker, AJCC: American Joint Committee on Cancer, UICC: International Union Against Cancer, IGCCCG: International Germ Cell Cancer Collaborative Group

## Diagnosis and surgical management

Testicular cancer commonly presents as a unilateral lump or painless swelling noticed incidentally. Pain is less common, with a third of patients presenting with a dull ache, and acute pain is uncommon, occurring in 10% of patients at presentation. Testis cancers uncommonly present with symptoms related to metastatic disease [3]. The clinical examination may uncover a testicular enlargement, and ultrasound examination confirms the existence of an intrascrotal tumor [5]. Pure testicular seminomas do not have specific serum tumor markers, but in certain cases can produce a small amount of βHCG (β-subunit of human chorionic gonadotropin) [6].

High inguinal orchiectomy is the standard initial treatment for suspected testicular carcinoma [7]. This strategy allows accurate staging and histological diagnosis of the tumor, while ensuring the best local control and minimizing treatment morbidity. Nonstandard surgical approaches (scrotal violations), including scrotal orchiectomy, open testicular biopsy and fine needle aspiration, have historically been condemned as significantly compromising prognosis. Patients with scrotal violation are often subjected to potentially morbid or disfiguring local therapies. In addition, patients with scrotal violations are usually disqualified from surveillance protocols [8].

Several groups have proposed organ-sparing orchiectomy as an alternative option for a small group of patients with bilateral testicular tumors, lesions in a solitary testis, or metachronous contralateral tumors. This approach allows endocrinological, fertility, and psychological advantages for the patient, especially in younger men [4]. The German Testicular Cancer Intergroup and others have reported prospective data on partial orchiectomy for GCT in a small subset of carefully selected patients with a solitary testis or bilateral testicular tumors [4]. Selection criteria in these studies included: organ-confined disease with no infiltration of the rete-testis; a mass of < 2 cm in order to preserve testosterone-producing parenchyma; a negative postresection biopsy of the tumor bed; and conditions of cold ischemia to preserve the function of Sertoli and Leydig cells. Heidenreich et al. have treated 73 patients with GCT with partial orchiectomy using these criteria. Among these, 17 were synchronous, 52 were metachronous and 4 occurred in a solitary testicle. After a median follow-up of 91 months, 98.6% of patients had no evidence of disease and one died of systemic tumor progression. The presence of carcinoma in situ was described in 82.3% of patients. Eighty-five percent of all patients had normal endogenous serum testosterone levels and did not need exogenous androgen replacement [4].

Anatomic studies and detailed mapping studies of retroperitoneal lymph node dissections have increased our understanding of testicular lymphatic drainage and have sharpened the focus of clinical staging and treatment by identifying the most likely sites of metastatic disease. The first echelon of lymph nodes draining the right testis is located in the inter-aortocaval region, followed by the precaval and pre-aortic nodes [6]. Regarding left-sided tumors, the first nodal stations include the pre-aortic and para-aortic lymph nodes, left renal hilar nodes followed by the inter-aortocaval nodes [6]. Contralateral spread is common with right-sided tumors but is rarely seen with left-sided tumors and is usually associated with bulky disease [9]. More caudal deposits of metastatic disease usually reflect retrograde spread to distal iliac and inguinal lymph nodes secondary to a large volume of disease and, more rarely, aberrant testicular lymphatic drainage.

Data comparing para-aortic nodal spread between seminomatous and nonseminomatous testicular tumors do not exist. From a theoretical point of view, we consider that the primary zone of spread of testis tumors is similar, and is not dependent on the histology [10]. In all cases, those nodal areas are in close proximity to the L1-L4 sympathetic roots of the superior hypogastric plexus. When oncologically possible, they should be spared at least unilaterally to preserve the ejaculation function. This goes against the ancient dogma that required a systematic and extended bilateral node dissection. Contrary to NSGCT, retroperitoneal lymph node dissection (RPLND) is no longer regarded as a valid therapeutic option in seminomas [11].

A good knowledge of the pathways of lymphatic nodal spread is essential for the radiation oncologist in the planning of the radiation treatment of the retroperitoneal region.

## Histology

Seminoma can be divided into three pathologic categories: classical, spermatocytic, and seminoma with syncytiocytotrophoblastic cells. The spermatocytic type is rare, occurs in older men, and may have a better prognosis. The classical and the syncytiocytotrophoblastic types of seminoma behave similarly, although the syncytiocytotrophoblastic subtype is associated with increased serum βhCG levels. Occasionally, seminoma may contain numerous mitotic figures. When three or more mitotic figures are identified per high power field throughout the tumor, it is designated as seminoma with high mitotic index or anaplastic seminoma.

Historically, anaplastic seminoma was thought to be a more aggressive subtype of seminoma but subsequent data failed to confirm this finding [12,13]. As an example, in a retrospective analysis of prognostic factors for relapse among 638 men with stage I seminoma, there was only a trend towards worse five-year relapse-free survival with anaplastic as compared to classical histology (83 *vs *71%, *p = 0.056*); in multivariate analysis, only tumor size and rete-testis invasion were significant predictors of outcome [12]. Most seminomas are confined to the testicle. Spread beyond the tunica into the spermatic cord occurs only in a minority of patients.

## Stage I seminoma

Seminoma patients with clinical stage I (about 85% of all stages) have a substantial risk of locoregional lymph node micrometastases with a 20% risk of disease progression if no adjuvant therapy is administered after orchiectomy. A primary tumor size of 4 cm or more and invasion of the rete testis have been identified as independent factors associated with an increased risk of relapse in multivariate analysis in many retrospective studies [11,12,14-16]. Some authors consider spread to the rete-testis as a negative prognostic factor [12,14,16] even it is not yet validated. The almost optimal cure rate in these patients is close to 100%, regardless of these features. This can be achieved with one of three treatment options: surveillance with treatment only in the case of relapse, adjuvant RT, or adjuvant single-agent carboplatin CHT [11,17,18]. With a cause-specific survival rate of 100%, the question is no longer 'how can the disease be cured?' but rather 'how can we retain this excellent cure rate with the least risk of short- and long-term consequences?'. Decisions regarding the management of stage I seminoma in any individual are thus complex, and we need to take into account concerns about long-term complications of RT and CHT, as well as the patient's ability to comply with intensive surveillance.

### Active surveillance

Surveillance policies offer the opportunity to detect relapsing patients early whilst avoiding the morbidities and risks of treatment for most [19]. No prospective studies exist comparing surveillance alone versus adjuvant treatment (RT or CHT). Several large prospective nonrandomized studies of surveillance have been conducted over the past 15 years. Reports have demonstrated the feasibility of surveillance protocols, particularly when associated with effective salvage regimens [19]. Retrospective series from the Royal Marsden Hospital London, from the Princess Margaret Hospital (PMH), Toronto, and from a national collaboration in Denmark, have all concluded that surveillance is a reasonable policy, albeit with some practical difficulties in view of the lack of sensitivity of specific serum markers [15,20,21]. Consensus guidelines accept surveillance as an option, which can be offered to stage I seminoma patients following orchiectomy [11]. A recent paper which analyses retrospectively a total of 649 patients reports the evolution of treatment with an increased use of active surveillance for stage I disease (545 patients) without deaths related to seminoma [22]. The predominant site of relapse is in the para-aortic lymph nodes and most patients are asymptomatic at the time of detection. In the DATECA (Danish Testicular Carcinoma Study Group) and in the PMH retrospective studies, 41 of 49 relapses (82%) and 54 of 67 relapses (89%) occurred in the para-aortic lymph nodes, respectively. Other sites of relapse included the pelvic lymph nodes (approximately 3% overall), and very rarely the inguinal nodes and the lungs [19,21].

Active surveillance permits avoiding development of a second malignancy which is a concern for anyone exposed to RT or CHT, especially in men with early stage seminoma who are expected to survive for decades following treatment [23-25]. Data on the association of infradiaphragmatic RT with subsequent cardiovascular disease are conflicting [26,27]. The largest study to date has included 40.576 testicular cancer survivors from 14 population-based tumor registries in Europe and North America [23]. More than 7800 were followed for 20 years and 2065 for 30 years. An increased risk of second solid cancers was seen among men treated with RT alone (RR 2.0), CHT alone (RR 1.8), and with both modalities (RR 2.9) [23,24]. Other rare complications may happen, such as renal artery stenosis after RT [28].

The main drawback of surveillance is the need for intensive follow-up and repeated imaging for at least 5 to 10 years after radical orchiectomy. Disadvantages include expensive imaging tests, radiation exposure, anxiety related to the risk of recurrence and the potential for patients to be noncompliant with follow-up [29,30]. While there is a high rate of cure for patients who experience recurrence and undergo definitive treatment [19], they are likely to require combination CHT, which has a greater toxicity risk than adjuvant treatment with RT or single-dose carboplatin [25]. There is no consensus regarding the optimum follow-up for these patients [12]. Currently, patients at PMH are followed up with regular physical examination, measurement of serum tumor markers, and imaging for retroperitoneal and chest disease. Patients are followed up at 4-monthly intervals for the first 3 years, 6-monthly intervals in years 4-7, and yearly intervals thereafter. At each visit, a CT scan of the abdomen and pelvis is performed. Chest x-rays are obtained at alternate visits. Serum tumor marker levels are measured at each visit for the first 3 years of surveillance [12]. Some clinicians feel that there is an unnecessary number of CT scans with this scheme. The healthy testis must be closely watched during follow-up, as the long-term risk of developing a contralateral testis cancer after a previous seminoma is about 2-5%. This usually occurs within the first 6 years and the risk decreases with time [31]. During clinical examination, the palpation of the testis must be systematic. Teaching of auto-palpation techniques is also interesting and efficient, and should be done whenever possible. In high-risk patients (fertility problems, testis atrophy, history of cryptorchidism, contralateral testis microcalcifications on the ultrasound), an annual doppler ultrasound exam can be advised to detect early relapse, and allows conservative treatment [32]. In the meanwhile, risk assessment for recurrence based on rete-testis involvement and tumor size is the best model until now. This model has never been validated independently, but we believe it can help us to assess risk of recurrence in our daily practice. In the context of potential risks and benefits of treatment, physicians should consult with the patient, and family if necessary, to determine the willingness and ability to adhere to a surveillance program. Patients and families should also be informed of the salvage treatment options and their potential risks.

### Radiation therapy

Seminoma cells are extremely radiosensitive, and radiation therapy has been widely used for more than 60 years, and has an excellent long-term track record. This modality is still a standard management in pure seminomas in the United States, and in Europe it is used quite often [33,34].

Historically, RT was delivered by a cobalt source using two parallel opposed anterior and posterior treatment fields, were defined with the help of bony landmarks. The dose was 30 Gy using 15 fractions. The treated areas were the para-aortic, homolateral external iliac nodes and the orchiectomy scar. This technique was known as the «dog-leg». The fields spread generally up to the superior aspect of D11 or D10 down to the inguinal ligament. This was the standard method until the beginning of the 1980's. Since the 1990's, following the low pelvic relapse rates reported in stage I tumors (less than 5%), the indication for pelvic irradiation was challenged [10,35,36]. The results of this new approach were excellent with a low pelvic relapse rate [37-39]. The reduced volume permits limiting the area, preserving the remaining testicular function and will hopefully decrease the secondary cancer rate [40,41]. However this strategy is still debated [42] in spite of the very well conducted randomized study by the Medical Research Council (MRC) Testicular Cancer Working Party. In this trial 478 patients were randomized between para-aortic and pelvic RT versus para-aortic RT alone. The dose was 30 Gy in 15 fractions in both arms. The relapse rate was 3.4% in the first group, and all recurrences were localized above the diaphragm versus 4% in the second group with 1.6% in the pelvis. Gastrointestinal side effects were less important in the second group. Patients with a scrotal, inguinal or pelvic surgical history were excluded from the trial [43,44]. A recent retrospective Australian study contradicts this irradiation option, arguing that despite the proven efficiency of the irradiation in the patients known to have had cryptorchidism, surveillance alone or chemotherapy are still valid options [44].

Looking for a way to combine those two treatment options, Thomas et al. have proposed a para-aortic and common iliac vessel (inferior limit at the acetabulum) irradiation field. This technique is used at the PMH [36], and allows the inclusion of most of these possible relapse regions [37].

However, we note that the irradiation of the para-aortic nodes alone yields good results and that the risk of nodal relapse exists but is quite low. We find that a clinical target volume (CTV) on the right side, comprising the paracaval, precaval and inter aortocaval nodes is justified. The left side should comprise, additionally, the latero-aortic, pre-aortic and left renal hilar nodes [6,7,10,35]. The inguinal orchiectomy scar and ipsilateral scrotal contents are not treated unless scrotal violation has occurred during surgery. We propose a planned target volume (PTV) as the CTV plus a 0.5 cm margin in all directions.

The optimal RT dose is also still a matter of controversy [45]. Generally, the recommended dose is between 25 and 30 Gy in 15 to 20 fractions. The MRC trial is the only randomized study that evaluates a dose de-escalation. It compared a dose of 20 Gy versus 30 Gy with conventional fractionation in 625 patients. Ten percent of the patients were treated with a « dog-leg » field, and 90% with para-aortic fields. The relapse rates after a median follow-up of a little more than five years were not significantly different. The 20 Gy arm showed a slightly lower acute toxicity rate (moderate asthenia 5% *vs *20%, work incapacity 28 *vs *46%). The only death due to the primary disease was in the 20 Gy arm [44].

Following this MRC publication, we also use the 20 Gy dose option with a two-week treatment time, which is now the standard in our institution.

The long term specific survival rate after RT reaches 100% and the disease free survival about 95-97% [15,43,46,47]. The RT regimen is well tolerated by most of the patients. The rare deaths in most of the series are usually due to intercurrent disease. In older studies where patients received prophylactic mediastinal and supraclavicular node irradiation, a significant number of deaths were due to secondary cancers and radiation-induced cardiac toxicity.

In single or multicenter studies with a sufficient number of patients, the relapse rates were below 5% and the relapses within the RT field were rare [19,43,48,49]. In those cases, a biopsy was needed to avoid missing a different histology, such as a nonseminomatous tumor. Relapses were located mostly in the mediastinum, the supraclavicular region and the lungs. The inguinal region was seldom involved (about 0.5%) and only in particular cases such as after a prior inguinal surgery [50]. In many ways, RT was a victim of its own success, because given the very high cure rates and the fact that many men were diagnosed with testicular cancer at a young age (< 30 years), patients lived long enough to develop late RT toxicities RT [51].

### Interest of new radiotherapy techniques

**Figure 1:**
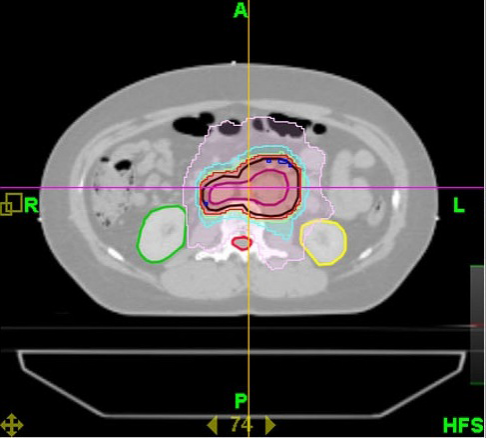
**Axial view showing planning target volume and isodose distribution using TomoTherapy, sparing kidneys and spinal cord in the case of stage I seminoma**.

In most of the old seminoma series, treatment was based on 2D irradiation techniques with cobalt machines and at higher doses compared to current recommendations. All this could be the cause of many short- and long-term complications [24,46,52-54]. The development of new medical imaging and exploration techniques has also greatly improved the quality of treatment. Currently, irradiation is delivered by a high-energy linear accelerator with a conformational technique, allowing the shaping of the treatment fields to the expected target volume which was planned with CT-scan images and 3D reconstruction (RT3D). With the help of multiple irradiation beams, this technique allows a better definition of the target volumes and a maximal sparing of the neighboring critical organs such as kidneys, spinal cord..... Computerized dosimetry and dose-volume histograms are now commonly used [55]. In our institution, we have treated several cases of seminoma by Intensity-Modulated Radiation Therapy (IMRT) (Figure 1). Many institutions tend to use the knowledge provided by functional imaging, associated with conventional imaging to better define the target volumes. Studies on the role of PET/CT (positron emission tomography coupled with a CT-scan) in the determination of treatment volumes are also under way [56].

### Chemotherapy

Cisplatin-based combination CHT is the gold standard in treating advanced testicular cancer, including both seminomatous and nonseminomatous tumors. Carboplatin is often preferred because of its better toxicity profile. In a phase II study, Oliver et al. were the first to describe the use of carboplatin in stage I seminomas. Seventy-eight patients were included (53 with two cycles, 25 with one), and after a 44-month median follow-up, only one relapse was observed [57]. In 2005, Oliver et al. reported the results of a multicenter randomized study. The latter included 1477 patients, and compared adjuvant para-aortic RT (para-aortic strip or dog-leg field and 20 or 30 Gy depending on the center) versus a single-cycle carboplatin CHT (area under curve (AUC) of 7 mg/ml/min) (Table 2) [58]. This non-inferiority trial showed a comparable 3-year disease-free survival time between both arms (94.8 *vs *95.9%; *p = 0.32*) [58]. These results were still comparable after a 6.5 years follow-up [18]. As a general rule, both treatments were well tolerated but with different toxicity profiles. With a little less asthenia, acute toxicity was somewhat less severe in the CHT arm. Long-term toxicity profiles are however, not yet available [58]. Only one seminoma-related death was recorded in the radiotherapy arm. Interestingly, there were significantly less contralateral germinal cell tumors in the CHT arm (*p = 0.003*). Among relapses, there was more para-aortic (74 *vs *9%) and pelvic nodal relapse (31 *vs *0%) in the para-aortic RT arm, showing the importance of RT in the prevention of those relapses [18,58]. One criticism about this study was that it was designed to exclude a 3% relapse risk in the carboplatin arm - it achieved an exclusion power of only 3.6% (95% confidence interval), the main goal consequently not being achieved.

**Table 2:** Relapses and survival in randomized controlled trials in stage 1 seminoma

Reference/No. of patients	Treatment	Total relapses	No. pelvic relapses	Relapse-free survival	Other
[44] n = 625	20 Gy RT (n = 313)	11	3	At 2 years: 97%	8/9 pelvic relapses occurred in the PA RT field group
		
	30 Gy RT (n = 312)	10	6	At 5 years: 97%*	

[43] n = 478	DL RT (n = 242)	9	0	At 3 years: 96.6% At 5 years: 96.2%*	3-years OS: 100%
	
	PA RT (n = 236)	9	4	At 3 years: 96% At 5 years: 96.1%*	3-years OS: 99.3%

[18,58] n = 1477	RT: PA or DL, 20 or 30 Gy (n = 904)	36	10	At 3 years: 95.9% At 5 years: 96%*	- All pelvic relapses occurred in the PA RT group - 74% of relapses in the carboplatin group occurred in the PA nodes
	
	1 cycle carboplatin (n = 573)	29	0	At 3 years: 94.8% At 5 years: 94.7%*	

RT: radiation therapy; DL: Dog-Leg; PA: para-aortic; OS: overall survival;* data retrieved in update.

There is a slightly increased relapse risk with the single-dose carboplatin regimen, as seen in one prospective study (9% *vs *0%) [17]. This was also the case in the update of the MRC/EORTC (European Organization for Research and Treatment of Cancer) trial [18].

Generally, the Calvert formula ("Calvert formula total carboplatin dose (mg) = (target AUC) × (GFR+25)") is used to find the optimal dose of carboplatin [59]. The calculated glomerular filtration rate (GFR) based on the blood creatinin level is often underestimated. This can lead to a wrong dose. The MRC/EORTC trial used a chromium-51 EDTA (Ethylenediaminetetraacetic acid) radio isotope clearance rate. This product is not Food and Drug Administration (FDA) authorized yet but other products exist today such as the iodine-125 iothalamate sodium or more simply the 24 hours proteinuria.

Current recommendation is to administer either a single carboplatin dose (that must be properly dosed with the help of renal scintigraphy) or two cycles spaced three weeks apart [34]. Although these recommendations are not yet supported by randomized studies, several phase II studies have evaluated the use of a 2-cycle carboplatin regimen. These results appear promising but longer follow-up and a phase III study are still needed.

Although the therapeutic equivalence between carboplatin and classic RT is well established, its long-term effects are still unknown. The sites of relapse after carboplatin and surveillance alone are comparable: both are often isolated and retroperitoneal [60].

In conclusion, the efficiency of the three different therapeutic options presented here seems to be equivalent. RT series, being the reference treatment, have a longer track record compared to the two newer options (Table 3, 4).

**Table 3:** Outcome of patients treated for seminoma from 1999 to 2008 [22]

Stage	Treatment	Number of patients	5-Year relapse rate (%)	Second relapse, n	5-Year disease-specific survival (%)	5-Year overall survival (%)	Dead of disease/treatment, n (%)	Death other cause, n (%)
	Surv	313	19.3	3^a ^(1%)	100	99	0	2 (1)
	
**I**	RT	159	2	0	100	99.3	0	1 (1)
	
	Carb	73	2	0	100	100	0	0

	RT	19	8.3	0	100	92.3	0	1 (3)
	
**II**	CHT	65	4.5	0	100	90.7	3 (5%)	2 (3)
	
	Other	3	0	0	100	100	0	0

**III**	CHT	17	0	0	100	100	0	0

a: After RT for first relapse.

Carb, single-agent carboplatin; CHT, primary combination chemotherapy; Other, other treatment modalities or combination of treatment modalities; RT, radiation; Surv, surveillance.

**Table 4:** Advantages and disadvantages of different management options in the treatment of stage I seminoma

Management option		Advantage	Disadvantage
**Surveillance**		- Excellent cancer cure rate - No treatment-related toxicity - Excellent salvage rate - Avoids overtreatment for most patients	- Frequent follow-up CT, with associated long-term risks - Anxiety related to the risk of recurrence

**Radiation therapy**	**Dog-leg**	- Excellent cancer cure rate - No need for routine CT - Lower recurrence rates compared with patients managed by surveillance	- Most patients are overtreated - Second malignancy risk - Cardiotoxicity - Fertility impairment

	**Para-aortic**	- Excellent cancer cure rate - Lower recurrence rate than patients managed by surveillance	- Frequent follow-up CT, with associated long-term risks - Second malignancy risk - Cardiotoxicity - Most patients are overtreated

**Chemotherapy**		- Excellent cancer cure rate - Lower acute toxicity than radiation therapy	- Long-term survival and toxicity unknown - Frequent follow-up CT, with associated long-term risks - Most patients are overtreated

CT: computerised tomography

## Stage II seminoma

Stage II seminoma are usually managed with RT or platinum-based combination CHT following orchiectomy. Obviously, surveillance is not an appropriate option for these men, and therapeutic RPLND has been largely replaced by CHT and/or RT [7]. No prospective randomized trial has been published to date for the treatment of stage II seminoma. The optimal treatment depends on the spread of lymph node invasion.

In old series, a difference between "bulky" and "non-bulky" disease was often made, but its precise definition varied between different centers. Mostly bulky disease was characterized by masses less than 7, most often, 5 cm in diameter. This corresponds to the latest TNM classification of N1-N2 and N3 stages (Table 1) [61].

After orchiectomy, the treatment of stage IIA and IIB seminomas with less than 2.5 cm nodal involvement (N2 < 2.5 cm) classically consists of RT which remains to this day the standard treatment [7,33]. These patients generally respond well to curative RT, and their clinical outcome is favorable in most cases. The need of chemotherapy for these patients is still questioned. Platinum-based CHT (PEB: cisplatin, etoposide, bleomycin for 3 cycles or PE: cisplatin, etoposide for 4 cycles, if there are arguments against bleomycin) were also used in some centers [7,33]. Prognosis remains good both with RT and CHT treatment. Five-year survival rates are about 95-100% [11,62-64].

Patients with more advanced disease with more than 2.5 cm nodes (IB stage with N2 between 2.5 and 5 or IIC stage) respond better to combined chemotherapy, despite a greater risk of toxicity compared to RT [65]. In these patients and in patients refusing RT, 3-4 cycles of PEB or PE CHT represents a valid option depending of the prognostic group [66]. Unlike stage I disease, a single agent carboplatin CHT is not proven to be efficient compared to combined cisplatin-based CHT [65].

A retrospective study by Domont et al., showed a significantly increased relapse rate after RT, especially with lymph nodes of more than 3 cm in diameter. Therefore, CHT plays an important role in stages IIB and beyond [67].

Ching et al., in a retrospective study including 79 cases, concluded with absence of proof for "prophylactic" left supraclavicular nodal RT; this volume of RT being of little use in 97% of the patients [68]. Mediastinal RT can be toxic for cardiac function, and was abandoned after the retrospective studies of Hanks et al. and Ledermann et al. [53,69]. Chung et al. recommend a classical infra-diaphragmatic RT including the para-aortic and same side (± contralateral) iliac nodes. Protection of the contralateral testis is fundamental to preserve fertility of often young patients. There is no proof that to include the contralateral iliac, inguinal, or scrotal regions in the RT volume is of any benefit. Scrotal irradiation was advised in the past in case of undescended testis, previous scrotal or inguinal surgery, or pT3 and pT4 tumors [11]. The role of the RT-CHT association is presently being evaluated [70].

In general, we have a longer follow-up with patients treated with RT than CHT especially with the newer drugs. Because of this, short term results can overestimate the true effect of the treatment.

In a phase II nonrandomized prospective study, Krege et al. showed that a monochemotherapy with carboplatin (AUC7) does not allow the full eradication of the retroperitoneal metastases in stage II seminomas [71]. Gilbert et al., in a letter to the editor, published results on 81 patients showing the superiority of RT given in association with carboplatin compared to RT alone [72]. This confirms the results of a previous study by Patterson et al. [64].

In stage IIA and IIB seminoma, the RT dose is between 30-36 Gy, depending on the size of the positive nodes [7]. The gross tumor volume (GTV) is defined on the planning CT-scan (computerised tomography). A first clinical target volume (CTV1) includes the GTV with a 0.5 cm margin, and a second (CTV2) includes the lymphatic risk areas (identical to CTV in stage I disease). We propose that the PTV should comprise both the CTV1 and CTV2 with a 0.5 cm margin [7,62-64,73]. In stage IIC Seminoma, although local control is possible with RT, there is a 50% risk of distant metastasis, and salvage may not be possible in all cases [74]. RT, therefore, has no major role in this stage of metastatic seminoma, as BEP combination CHT cures 95% of patients [75]. In addition, RT to the involved fields after CHT has not been shown in a retrospective analysis to add any survival benefit [76]. Today BEP CHT, as in more advanced stages, remains the standard of care, but increasing attention has been given to late toxicity of the treatment, and there is increased interest in further studies of a single agent CHT [77].

## Stage III seminoma

Most of the studies on advanced germinal cancer include both seminoma and nonseminomatous tumors [78]. There is no evidence that their chemosensitivity is any different [79,80]. As there is no bad prognostic subtype for advanced pure seminomas, most of the centers tend to treat them in the same way as the bad prognostic subtypes of nonseminoma. The current standard treatment consists of 3-4 cycles of BEP or EP CHT [5,34]. The most recent European consensus evaluates the risk of complications [11]. The retrospective Dutch study of Belt-Dusebout et al. establishes the risk of secondary cancer and cardiovascular complications following the treatment of testicular cancer in general and after CHT in particular [81]. Cisplatin dose-intensified CHT does not seem to be superior to standard BEP or RT [82]. Post therapeutic follow-up modalities consist of a four-week post CHT thoraco-abdomino-pelvic CT-scan [5]. The subsequent management depends on the size of the residual mass. If the latter is less than 3 cm in diameter, a simple surveillance in advised. If it is larger, a PET/CT exam is recommended. If the latter remains positive, a definitive confirmation by biopsy is necessary. If the PET/CT is negative, surveillance may be sufficient [33,83]. In the presence of active residual tumoral tissue, RT or CHT remains the treatment of choice [5,33].

## Management of relapse

Treatment of relapse depends on different parameters such as the nature of the initial treatment and the subsequent response, the localization, and the time since treatment. Most of the stage I seminoma patients who are under surveillance can be salvaged by RT or cisplatin CHT alone. Surgery is not an option [14,21].

In case of relapse after RT, it will almost always be outside the previous treatment volume. The recommended treatment scheme is a CHT identical to that used in stage IIC and III, which is efficient in the majority of the patients [7]. Reirradiation is also possible if the relapse is late, localized, and represent a small volume, such as a solitary adenopathy. In this case, some groups recommend pelvic nodal dissection [84]. Our opinion is that it is by far not the best option in stage I disease relapse.

In the case of relapse after CHT, and if it occurs less than three months after one CHT cycle, the disease is still considered to be sensitive to a platinum-based CHT salvage treatment [7]. The chemosensitivity persists even after the second or third CHT cycles [58]. Cisplatin is the fundamental drug that must be part of any salvage CHT [77,85]. The most used first line salvage protocols are the VIP (cisplatin, etoposide, ifosfamide), TIP (paclitaxel, ifosfamide, cisplatin) or VeIP (vinblastine, ifosfamide, cisplatin) schedules [84]. In fact, relapse after a platinum-based CHT is very rare, and about 50% of them are cured by a salvage CHT [84,86].

Dose-intensification CHT has not been shown to be of any interest in first or second line salvage treatments [82]. Post CHT salvage surgery must not be used too promptly, as a retarded regression of the residual masses is frequently observed [83,87]. Surgery can possibly be considered in case of CHT failure, although it is mainly used in nonseminomatous tumors [88,89].

Up to now, surgery has only been considered only for residual masses over 3 cm. The use of FDG-PET/CT (18F-fluorodeoxyglucose positron emission tomography) scanning allowed a change in this recommendation [90]. An FDG-PET/CT fixing lesion can be surgically removed. Even if technically difficult, resection must be complete.

In patients resistant to CHT, such as those who have never normalized their markers after a first course of CHT or who have not responded to salvage CHT, there is presently no standard treatment. Some authors advise the use of new drugs such as gemcitabine and paclitaxel. Dose-intensification CHT is under investigation. The place of surgery has yet to be defined [91].

## βHCG secreting seminoma

βHCG secreting seminoma is a rare form of pure seminoma with an incidence of about 10-20% [92]. An increase in serum βhCG primarily reflects higher tumor burden but not necessarily a greater metastatic potential [93]. It has the same clinical and evolutive characteristics as the non-secreting seminoma. Its treatment is controversial but two studies have been able to determine that its prognosis is identical to that of the non-secreting form [94,95]. In stage I, the concentration of this glycoprotein should return to normal after surgery [95]. If not, it is strongly suggestive of disease of at least stage II [94]. In this case, the blood level of βHCG should be normalized after an adjuvant RT or CHT treatment [95].

In older series, stage I βHCG secreting seminoma was considered to carry a worse prognosis [96]. This was probably due to a selection bias. Even if a βHCG level is associated with a more important tumor volume, recent series report a prognosis comparable to the non-secreting forms [94,95,97,98]. The experience in stage II secreting seminomas is more limited. The treatment is also generally the same as that for non-secreting seminomas. The prognosis is independent of the βHCG blood level [92,94,95,99].

## Conclusion

Pure seminoma is a rare pathology of the young adult in whom it is often discovered in the early stages. Its prognosis is generally excellent. Many therapeutic options are available, especially in stage I tumors. Disease control and survival rates are similar. To choose the best therapeutic option, the physician must consider the economic impact, the psychological profile of the patient and future compliance to treatment. All the options must be presented to the patient, so that he can give his informed consent. A randomized study comparing the three therapeutic options is needed from an academic standpoint knowing its difficult concretization in practice. Although patients managed with surveillance have a higher relapse rate, survival is likely equivalent regardless of initial management because of excellent salvage treatment. For advanced stages, the treatment includes RT, but the mainstay is platinum based CHT: Trials on combined RT and CHT are under way. βHCG secreting seminoma is a rare form of pure seminoma, and its prognosis and treatment is comparable to those of non-secreting seminoma. In case of relapse, salvage options depend on previous treatments. Presently, FDG-PET/CT is an important imaging modality in the therapeutic decision in case of post CHT residual masses. Dose-intensification CHT regimens are still being investigated.

## Competing interests

The authors declare that they have no competing interests.

## Authors' contributions

NB, AZ: conception and design. NB drafted the manuscript, AC, NB, KK, SB, EH, ROM, MO and AZ criticized the manuscript. All authors read and approved the final manuscript.

## References

[B1] Jemal A Siegel R Xu J Ward E Cancer statistics, 2010 CA Cancer J Clin 2010 60 5 277 300 10.3322/caac.20073 20610543

[B2] Levi F Te VC Randimbison L La Vecchia C Trends in testicular cancer incidence in Vaud, Switzerland Eur J Cancer Prev 2003 12 4 347 9 10.1097/00008469-200308000-00017 12883390

[B3] Khan O Protheroe A Testis Cancer Postgrad Med J 2007 83 984 624 32 10.1136/pgmj.2007.057992 17916870 PMC2600126

[B4] Heidenreich A Weissbach L Höltl W Albers P Kliesch S Köhrmann KU DIeckmann KP German Testicular Cancer Study Group German Testicular Cancer Study Group. Organ sparing surgery for malignant germ cell tumor of the testis J Urol 2001 166 6 2161 5 10.1016/S0022-5347(05)65526-7 11696727

[B5] Mottet N Culine S Iborra F Avances C Bastide C Lesourd A Michel F Rigaud J Testicular tumors Prog Urol 2007 17 6 1035 45 10.1016/S1166-7087(07)74779-6 18153986

[B6] Donohue JP Zachary JM Maynard BR Distribution of nodal metastases in non seminomatous testis cancer J Urol 1982 128 315 20 7109099 10.1016/s0022-5347(17)52904-3

[B7] Schmoll HJ Jordan K Huddart R Pes MP Horwich A Fizazi K Kataja V ESMO Guidelines Working Group Testicular seminoma: ESMO Clinical Practice Guidelines for diagnosis, treatment and follow-up Ann Oncol 2010 21 5 140 6 10.1093/annonc/mdq176 19887468

[B8] Capelouto CC Clark PE Ransil BJ Loughlin KR A review of scrotal violation in testicular cancer: is adjuvant local therapy necessary? J Urol 1995 153 3 Pt 2 981 5 7853587

[B9] Pizzocaro G Nicolai N Salvioni R Evolution of the management of stage I nonseminomatous germ-cell tumors of the testis World J Urol 1994 12 3 113 9 7524920 10.1007/BF00192265

[B10] Kiricuta IC Sauer J Bohndorf W Omission of the pelvic irradiation in Stage I testicular seminoma: A study of postorchiectomy paraaortic radiotherapy Int J Radiat Oncol Biol Phys 1996 35 2 293 8 10.1016/0360-3016(96)00093-4 8635936

[B11] Krege S Beyer J Souchon R Albers P Albrecht W Algaba F Bamberg M Bodrogi I Bokemeyer C Cavallin-Ståhl E Classen J Clemm C Cohn-Cedermark G Culine S Daugaard G De Mulder PH De Santis M de Wit M de Wit R Derigs HG Dieckmann KP Dieing A Droz JP Fenner M Fizazi K Flechon A Fosså SD del Muro XG Gauler T Geczi L European consensus conference on diagnosis and treatment of germ cell cancer: a report of the second meeting of the European Germ Cell Cancer Consensus group (EGCCCG): part I Eur Urol 2008 53 3 478 96 10.1016/j.eururo.2007.12.024 18191324

[B12] Warde P Specht L Horwich A Oliver T Panzarella T Gospodarowicz M von der Maase H Prognostic factors for relapse in stage I seminoma managed by surveillance: a pooled analysis J Clin Oncol 2002 20 22 4448 52 10.1200/JCO.2002.01.038 12431967

[B13] Bobba VS Mittal BB Hoover SV Kepka A Classical and anaplastic seminoma: difference in survival Radiology 1988 167 3 849 52 3129752 10.1148/radiology.167.3.3129752

[B14] Warde P Gospodarowicz MK Banerjee D Panzarella T Sugar L Catton CN Sturgeon JF Moore M Jewett MA Prognostic factors for relapse in stage I testicular seminoma treated with surveillance J Urol 1997 157 5 1705 9 10.1016/S0022-5347(01)64839-0 9112510

[B15] Warde P Gospodarowicz MK Panzarella T Catton CN Sturgeon JF Moore M Goodman P Jewett MA Stage I testicular seminoma: results of adjuvant irradiation and surveillance J Clin Oncol 1995 13 9 2255 62 7666083 10.1200/JCO.1995.13.9.2255

[B16] Kamba T Kamoto T Okubo K Teramukai S Kakehi Y Matsuda T Ogawa O Outcome of different post-orchiectomy management for stage I seminoma: Japanese multi-institutional study including 425 patients Int J Urol 2010 17 12 980 7 10.1111/j.1442-2042.2010.02645.x 20955354 PMC3017741

[B17] Dieckmann KP Brüggeboes B Pichlmeier U Küster J Müllerleile U Bartels H Adjuvant treatment of clinical stage I seminoma: is a single course of carboplatin sufficient? Urology 2000 55 1 102 6 10.1016/S0090-4295(99)00376-3 10654903

[B18] Oliver RT Mead GM Fogarty PJ Stenning SP MRC TE19 and EORTC 30982 trial collaborators Radiotherapy versus carboplatin for stage I seminoma: Updated analysis of the MRC/EORTC randomized trial (abstract) Proc Am Soc Clin Oncol 2008 26 1 (Part II, 1006s)

[B19] Warde PR Chung P Sturgeon J Panzarella T Giuliani M Tew-George B Jewett M Bayley A Moore M Catton C Gospodarowicz M Should surveillance be considered the standard of care in stage I seminoma? J: Clin Oncol (Meeting Abstracts) 2005 23 4520

[B20] Horwich A Alsanjari N A'Hern R Nicholls J Dearnaley DP Fisher C Surveillance following orchidectomy for stage I testicular Seminoma Br J Cancer 1992 65 5 775 8 10.1038/bjc.1992.164 1586607 PMC1977398

[B21] von der Maase H Specht L Jacobsen GK Jakobsen A Madsen EL Pedersen M Rørth M Schultz H Surveillance following orchidectomy for stage I seminoma of the testis Eur J Cancer 1993 29A 14 1931 4 8280484 10.1016/0959-8049(93)90446-m

[B22] Kollmannsberger C Tyldesley S Moore C Chi KN Murray N Daneshmand S Black P Duncan G Hayes-Lattin B Nichols C Evolution in management of testicular seminoma: population-based outcomes with selective utilization of active therapies Ann Oncol 2011 22 4 808 14 10.1093/annonc/mdq466 20926549

[B23] Travis LB Curtis RE Storm H Hall P Holowaty E Van Leeuwen FE Kohler BA Pukkala E Lynch CF Andersson M Bergfeldt K Clarke EA Wiklund T Stoter G Gospodarowicz M Sturgeon J Fraumeni JF Jr Boice JD Jr Risk of second malignant neoplasms among long-term survivors of testicular cancer J Natl Cancer Inst 1997 89 19 1429 39 10.1093/jnci/89.19.1429 9326912

[B24] Travis LB Fosså SD Schonfeld SJ McMaster ML Lynch CF Storm H Hall P Holowaty E Andersen A Pukkala E Andersson M Kaijser M Gospodarowicz M Joensuu T Cohen RJ Boice JD Jr Dores GM Gilbert ES Second cancers among 40,576 testicular cancer patients: focus on long-term survivors J Natl Cancer Inst 2005 97 18 1354 65 10.1093/jnci/dji278 16174857

[B25] Powles T Robinson D Shamash J Moller H Tranter N Oliver T The long-term risks of adjuvant carboplatin treatment for stage I seminoma of the testis Ann Oncol 2008 19 3 443 7 18048383 10.1093/annonc/mdm540

[B26] Huddart RA Norman A Shahidi M Horwich A Coward D Nicholls J Dearnaley DP Cardiovascular disease as a long-term complication of treatment for testicular cancer J Clin Oncol 2003 21 8 1513 23 10.1200/JCO.2003.04.173 12697875

[B27] Haugnes HS Aass N Fosså SD Dahl O Klepp O Wist EA Wilsgaard T Bremnes RM Predicted cardiovascular mortality and reported cardiovascular morbidity in testicular cancer survivors J Cancer Surviv 2008 2 3 128 37 10.1007/s11764-008-0054-1 18792787

[B28] Mulla MG Ananthkrishnan G Mirza MS Bungay P Puri S Chakraborti P Renal artery stenosis after radiotherapy for stage I seminoma, a case report Clin Oncol (R Coll Radiol) 2007 19 3 209 10.1016/j.clon.2007.01.114 17359909

[B29] Sharda NN Kinsella TJ Ritter MA Adjuvant radiation versus observation: a cost analysis of alternate management schemes in early-stage testicular seminoma J Clin Oncol 1996 14 11 2933 9 8918490 10.1200/JCO.1996.14.11.2933

[B30] Buchholz TA Walden TL Prestidge BR Cost-effectiveness of posttreatment surveillance after radiation therapy for early stage seminoma Cancer 1998 82 6 1126 33 10.1002/(SICI)1097-0142(19980315)82:6<1126::AID-CNCR17>3.0.CO;2-8 9506359

[B31] Fosså SD Chen J Schonfeld SJ Risk of contralateral testicular cancer: a population-based study of 29,515 U:S: men J Natl Cancer Inst 2005 97 14 1056 66 10.1093/jnci/dji185 16030303

[B32] Culine S Michel F Rocher L Mottet N Davin JL Comité de Cancérologie de l'Association Française d'Urologie. Follow-up of testicular germ cell tumours. Guidelines of the Comité de Cancérologie de l'Association Française d'Urologie Prog Urol 2005 15 4 593 6 16459667

[B33] Krege S Beyer J Souchon R Albers P Albrecht W Algaba F Bamberg M Bodrogi I Bokemeyer C Cavallin-Ståhl E Classen J Clemm C Cohn-Cedermark G Culine S Daugaard G De Mulder PH De Santis M de Wit M de Wit R Derigs HG Dieckmann KP Dieing A Droz JP Fenner M Fizazi K Flechon A Fosså SD del Muro XG Gauler T Geczi L European consensus conference on diagnosis and treatment of germ cell cancer: a report of the second meeting of the European Germ Cell Cancer Consensus group (EGCCCG): part II: Eur Urol 2008 53 3 497 513 10.1016/j.eururo.2007.12.025 18191015

[B34] National Comprehensive Cancer Network clinical practice guidelines in oncology testicular cancer V:1; 2011 http://www.nccn.org/professionals/physician_gls/f_guidelines.asp 10.6004/jnccn.2009.0047 19555582

[B35] Hunter M Peschel RE Testicular seminoma. Results of the Yale University experience, 1964-1984 Cancer 1989 64 8 1608 11 10.1002/1097-0142(19891015)64:8<1608::AID-CNCR2820640809>3.0.CO;2-1 2790671

[B36] Thomas GM Is "optimal" radiation for stage I seminoma yet defined? J Clin Oncol 1999 17 9 3004 5 10561381

[B37] Classen J Schmidberger H Meisner C Winkler C Dunst J Souchon R Weissbach L Budach V Alberti W Bamberg M German Testicular cancer study Group (GTCSG) German Testicular cancer study Group Para-aortic irradiation for stage I testicular seminoma: results of a prospective study in 675 patients. A trial of the German testicular cancer study Br J Cancer 2004 90 12 2305 11 15150576 10.1038/sj.bjc.6601867 PMC2409532

[B38] Logue JP Harris MA Livsey JE Swindell R Mobarek N Read G Short course para-aortic radiation for stage I seminoma of the testis Int J Radiat Oncol Biol Phys 2003 57 5 1304 9 10.1016/S0360-3016(03)00754-5 14630266

[B39] Melchior D Hammer P Fimmers R Schüller H Albers P Long term results and morbidity of paraaortic compared with paraaortic and iliac adjuvant radiation in clinical stage I seminoma Anticancer Res 2001 21 2989 93 11712799

[B40] Zwahlen DR Martin JM Millar JL Schneider U Effect of radiotherapy volume and dose on secondary cancer risk in stage I testicular seminoma Int J Radiat Oncol Biol Phys 2008 70 3 853 8 10.1016/j.ijrobp.2007.10.007 18164856

[B41] Jacobsen KD Olsen DR Fosså K Fosså SD External beam abdominal radiotherapy in patients with seminoma stage I: field type, testicular dose, and spermatogenesis Int J Radiat Oncol Biol Phys 1997 38 1 95 102 10.1016/S0360-3016(96)00597-4 9212009

[B42] Power RE Kennedy J Crown J Fraser I Thornhill JA Pelvic recurrence in stage I seminoma: a new phenomenon that questions modern protocols for radiotherapy and follow-up Int J Urol 2005 12 4 378 82 10.1111/j.1442-2042.2005.01114.x 15948726

[B43] Fosså SD Horwich A Russell JM Roberts JT Cullen MH Hodson NJ Jones WG Yosef H Duchesne GM Owen JR Grosch EJ Chetiyawardana AD Reed NS Widmer B Stenning SP Optimal planning target volume for stage I testicular seminoma: A Medical Research Council randomized trial. Medical Research Council Testicular Tumor J Clin Oncol 1999 17 4 1146 10561173 10.1200/JCO.1999.17.4.1146

[B44] Jones WG Fossa SD Mead GM Roberts JT Sokal M Horwich A Stenning SP Randomized trial of 30 versus 20 Gy in the adjuvant treatment of stage I Testicular Seminoma: a report on Medical Research Council Trial TE18, European organisation for the research and treatment of cancer trial 30942 (ISRCTN18525328) J Clin Oncol 2005 23 6 1200 8 10.1200/JCO.2005.08.003 15718317

[B45] Milosevic MF Gospodarowicz M Warde P Management of testicular seminoma Semin Surg Oncol 1999 17 4 240 9 10.1002/(SICI)1098-2388(199912)17:4<240::AID-SSU4>3.0.CO;2-Q 10588852

[B46] Fosså SD Aass N Kaalhus O Radiotherapy for testicular seminoma stage I: treatment results and long-term post-irradiation morbidity in 365 patients Int J Radiat Oncol Biol Phys 1989 16 2 383 8 10.1016/0360-3016(89)90334-9 2921142

[B47] Oldenburg J Martin JM Fosså SD Late relapses of germ cell malignancies - incidence, management, and prognosis J Clin Oncol 2006 24 35 5503 11 10.1200/JCO.2006.08.1836 17158535

[B48] Coleman JM Coleman RE Turner AR Radstone CR Champion AE The management and clinical course of testicular seminoma: 15 years' experience at a single institution Clin Oncol (R Coll Radiol) 1998 10 4 237 41 10.1016/s0936-6555(98)80007-1 9764375

[B49] Santoni R Barbera F Bertoni F De Stefani A Livi L Paiar F Scoccianti S Magrini SM Stage I seminoma of the testis: a bi-institutional retrospective analysis of patients treated with radiation therapy only BJU Int 2003 92 1 47 52 10.1046/j.1464-410X.2003.04273.x 12823382

[B50] Borge N Fosså SD Ous S Stenwig AE Lien HH Late recurrence of testicular cancer J Clin Oncol 1988 6 8 1248 53 2842463 10.1200/JCO.1988.6.8.1248

[B51] Hoffman KE Chen MH Punglia RS Beard CJ D'Amico AV Influence of year of diagnosis, patient age, and sociodemographic status on recommending adjuvant radiation treatment for stage I testicular seminoma J Clin Oncol 2008 26 24 3937 42 10.1200/JCO.2008.16.5043 18711182 PMC2867767

[B52] Fosså SD Oldenburg J Dahl AA Short- and long-term morbidity after treatment for testicular cancer BJU Int 2009 104 9 Pt B 1418 22 19840023 10.1111/j.1464-410X.2009.08869.x

[B53] Hanks GE Peters T Owen J Seminoma of the testis: Long-term beneficial and deleterious effects of radiation Int J Radiat Oncol Biol Phys 1992 24 913 919 10.1016/0360-3016(92)90475-W 1447034

[B54] Richiardi L Scélo G Boffetta P Hemminki K Pukkala E Olsen JH Weiderpass E Tracey E Brewster DH McBride ML Kliewer EV Tonita JM Pompe-Kirn V Kee-Seng C Jonasson JG Martos C Brennan P Second malignancies among survivors of germ-cell testicular cancer: a pooled analysis between 13 cancer registries Int J Cancer 2007 120 3 623 31 10.1002/ijc.22345 17096341

[B55] Martin JM Joon DL Ng N Grace M Gelderen DV Lawlor M Wada M Joon ML Quong G Khoo V Towards individualised radiotherapy for Stage I seminoma Radiother Oncol 2005 76 3 251 6 10.1016/j.radonc.2005.08.005 16169622

[B56] Zouhair A Ozsahin M Schaffer M Albrecht S Camus F Jichlinski P Mirimanoff RO Bischof Delaloye A Meuwly JY Prior JO Positron Emission Tomography and Computer Tomography (PET/CT) in Prostate, Bladder, and Testicular Cancers Curr Med Chem 2010 17 23 2492 502 10.2174/092986710791556014 20491646

[B57] Oliver RT Edmonds PM Ong JY Ostrowski MJ Jackson AW Baille-Johnson H Williams MV Wiltshire CR Mott T Pratt WR Pilot studies of 2 and 1 course carboplatin as adjuvant for stage I seminoma: should it be tested in a randomized trial against radiotherapy? Int J Radiat Oncol Biol Phys 1994 29 1 3 8 10.1016/0360-3016(94)90219-4 8175442

[B58] Oliver RT Mason MD Mead GM von der Maase H Rustin GJ Joffe JK de Wit R Aass N Graham JD Coleman R Kirk SJ Stenning SP MRC TE19 collaborators and the EORTC 30982 collaborators Radiotherapy versus single-dose carboplatin in adjuvant treatment of stage I seminoma: a randomised trial Lancet 2005 366 9482 293 300 10.1016/S0140-6736(05)66984-X 16039331

[B59] Calvert AH Egorin MJ Carboplatin dosing formulae: gender bias and the use of creatinine-based methodologies Eur J Cancer 2002 38 1 11 6 10.1016/S0959-8049(01)00340-9 11750834

[B60] Mead GM Fossa SD Oliver RT Fogarty PJ Pollock P Stenning SP Relapse patterns in 2,466 stage 1 seminoma patients (pts) entered into Medical Research Council randomised trials J Clin Oncol (Meeting Abstracts) 2008 26 5020

[B61] AJCC Cancer Staging Manual Ann Oncol 2010 21 6 5 Springer Verlag New York 140 6 10.1093/annonc/mdq176 19887468

[B62] Schmidberger H Bamberg M Meisner C Classen J Winkler C Hartmann M Templin R Wiegel T Dornoff W Ross D Thiel HJ Martini C Haase W Radiotherapy in stage IIA and IIB testicular seminoma with reduced portals: a prospective multicenter study Int J Radiat Oncol Biol Phys 1997 39 2 321 6 9308934 10.1016/s0360-3016(97)00155-7

[B63] Classen J Schmidberger H Meisner C Souchon R Sautter-Bihl ML Sauer R Weinknecht S Köhrmann KU Bamberg M Radiotherapy for stages IIA/B testicular seminoma: final report of a prospective multicenter clinical trial J Clin Oncol 2003 21 6 1101 6 10.1200/JCO.2003.06.065 12637477

[B64] Patterson H Norman AR Mitra SS Nicholls J Fisher C Dearnaley DP Horwich A Mason MD Huddart RA Combination carboplatin and radiotherapy in the management of stage II testicular seminoma: comparison with radiotherapy treatment alone Radiother Oncol 2001 59 1 5 11 10.1016/S0167-8140(00)00240-1 11295200

[B65] Arranz Arija JA García del Muro X Gumà J Aparicio J Salazar R Saenz A Carles J Sánchez M Germà-Lluch JR E400P in advanced seminoma of good prognosis according to the international germ cell cancer collaborative group (IGCCCG) classification: the Spanish Germ Cell Cancer Group experience Ann Oncol 2001 12 4 487 91 10.1023/A:1011127715764 11398880

[B66] Einhorn LH Williams SD Loehrer PJ Birch R Drasga R Omura G Greco FA Evaluation of optimal duration of chemotherapy in favorable-prognosis disseminated germ cell tumors: a Southeastern Cancer Study Group protocol J Clin Oncol 1989 7 3 387 91 2465391 10.1200/JCO.1989.7.3.387

[B67] Domont J Laplanche A de Crevoisier R Theodore C Wibault P Fizazi K A risk-adapted strategy of radiotherapy and cisplatin-based chemotherapy in stage II seminoma: results of a 20-year experience (abstract) J Clin Oncol 2005 23 16 4571

[B68] Chung PW Warde PR Panzarella T Bayley AJ Catton CN Milosevic MF Jewett MA Sturgeon JF Moore M Gospodarowicz MK Appropriate radiation volume for stage IIA/B testicular seminoma Int J Radiat Oncol Biol Phys 2003 56 3 746 8 10.1016/S0360-3016(03)00011-7 12788180

[B69] Lederman GS Sheldon TA Chaffey JT Herman TS Gelman RS Coleman CN Cardiac disease after mediastinal irradiation for seminoma Cancer 1987 60 4 772 6 10.1002/1097-0142(19870815)60:4<772::AID-CNCR2820600411>3.0.CO;2-A 3594400

[B70] von der Maase H Do we have a new standard of treatment for patients with seminoma stage IIA and stage IIB? Radiother Oncol 2001 59 1 1 3 10.1016/S0167-8140(01)00344-9 11295199

[B71] Krege S Boergermann C Baschek R Hinke A Pottek T Kliesch S Dieckmann KP Albers P Knutzen B Weinknecht S Schmoll HJ Beyer J Ruebben H German Testicular Cancer Study Group (GTCSG) German Testicular Cancer Study Group (GTCSG). Single agent carboplatin for CS IIA/B testicular seminoma. A phase II study of the German Testicular Cancer Study Group (GTCSG) Ann Oncol 2006 17 2 276 80 16254023 10.1093/annonc/mdj039

[B72] Gilbert DC Pudney D Van As N Dearnaley D Horwich A Huddart R Early outcomes of treating stage IIA/B seminoma with a single cycle of carboplatin and radiotherapy (abstract) J Clin Oncol 2009 27 12 2101 2 10.1200/JCO.2008.21.5269 19289608

[B73] Jacobsen GK Mellemgaard A Engelholm SA Moller H Increased incidence of sarcoma in patients treated for testicular seminoma Eur J Cancer 1993 29A 664 8 8471322 10.1016/s0959-8049(05)80342-9

[B74] Warde P Gospodarowicz M Panzarella T Catton C Sturgeon J Moore M Jewett M Management of stage II seminoma J Clin Oncol 1998 16 1 290 4 9440755 10.1200/JCO.1998.16.1.290

[B75] Fosså SD Oliver RT Stenning SP Horwich A Wilkinson P Read G Mead GM Roberts JT Rustin G Cullen MH Kaye SB Harland SJ Cook P Prognostic factors for patients with advanced seminoma treated with platinum-based chemotherapy Eur J Cancer 1997 33 9 1380 7 10.1016/S0959-8049(96)00425-X 9337678

[B76] Duchesne GM Stenning SP Aass N Mead GM Fosså SD Oliver RT Horwich A Read G Roberts IT Rustin G Cullen MH Kaye SB Harland SJ Cook PA Radiotherapy after chemotherapy for metastatic seminoma--a diminishing role. MRC Testicular Tumour Working Party Eur J Cancer 1997 33 6 829 35 10.1016/S0959-8049(97)00033-6 9291801

[B77] Bokemeyer C Kollmannsberger C Stenning S Hartmann JT Horwich A Clemm C Gerl A Meisner C Rückerl CP Schmoll HJ Kanz L Oliver T Metastatic seminoma treated with either single agent carboplatin or cisplatin-based combination chemotherapy: a pooled analysis of two randomised trials Br J Cancer 2004 91 4 683 7 15266338 10.1038/sj.bjc.6602020 PMC2364800

[B78] Nichols CR Catalano PJ Crawford ED Vogelzang NJ Einhorn LH Loehrer PJ Randomized comparison of cisplatin and etoposide and either bleomycin or ifosfamide in treatment of advanced disseminated germ cell tumors: an Eastern Cooperative Oncology Group, Southwest Oncology Group, and Cancer and Leukemia Group B Study J Clin Oncol 1998 16 4 1287 93 9552027 10.1200/JCO.1998.16.4.1287

[B79] Gholam D Fizazi K Terrier-Lacombe MJ Jan P Culine S Theodore C Advanced seminoma-treatment results and prognostic factors for survival after first-line, cisplatin-based chemotherapy and for patients with recurrent disease: a single-institution experience in 145 patients Cancer 2003 98 4 745 52 10.1002/cncr.11574 12910518

[B80] Mencel PJ Motzer RJ Mazumdar M Vlamis V Bajorin DF Bosl GJ Advanced seminoma: treatment results, survival, and prognostic factors in 142 patients J Clin Oncol 1994 12 1 120 6 7505805 10.1200/JCO.1994.12.1.120

[B81] van den Belt-Dusebout AW de Wit R Gietema JA Horenblas S Louwman MW Ribot JG Hoekstra HJ Ouwens GM Aleman BM van Leeuwen FE Treatment-specific risks of second malignancies and cardiovascular disease in 5-year survivors of testicular cancer J Clin Oncol 2007 25 28 4370 8 10.1200/JCO.2006.10.5296 17906202

[B82] Giannis M Aristotelis B Vassiliki K Ioannis A Konstantinos S Nikolaos A Georgios P Georgios P Pantelis P Meletios-Athanasios D Cisplatin-based chemotherapy for advanced seminoma: report of 52 cases treated in two institutions J Cancer Res Clin Oncol 2009 135 11 1495 500 10.1007/s00432-009-0596-2 19437039 PMC12160265

[B83] Flechon A Bompas E Biron P Droz JP Management of post-chemotherapy residual masses in advanced seminoma J Urol 2002 168 5 1975 9 10.1016/S0022-5347(05)64275-9 12394688

[B84] Miller KD Loehrer PJ Gonin R Einhorn LH Salvage chemotherapy with vinblastine, ifosfamide, and cisplatin in recurrent seminoma J Clin Oncol 1997 15 4 1427 31 9193335 10.1200/JCO.1997.15.4.1427

[B85] Culine S Abs L Terrier-Lacombe MJ Théodore C Wibault P Droz JP Cisplatin-based chemotherapy in advanced seminoma: the Institut Gustave Roussy experience Eur J Cancer 1998 34 3 353 8 9640221 10.1016/s0959-8049(97)10070-3

[B86] Vuky J Tickoo SK Sheinfeld J Bacik J Amsterdam A Mazumdar M Reuter V Bajorin DF Bosl GJ Motzer RJ Salvage chemotherapy for patients with advanced pure seminoma J Clin Oncol 2002 20 1 297 301 10.1200/JCO.20.1.297 11773182

[B87] Puc HS Heelan R Mazumdar M Herr H Scheinfeld J Vlamis V Bajorin DF Bosl GJ Mencel P Motzer RJ Management of residual mass in advanced seminoma: results and recommendations from the Memorial Sloan-Kettering Cancer Center J Clin Oncol 1996 14 2 454 60 8636757 10.1200/JCO.1996.14.2.454

[B88] Motzer RJ Bosl GJ Geller NL Penenberg D Yagoda A Golbey R Whitmore WF Jr Fair WR Sogani P Herr H Advanced seminoma: the role of chemotherapy and adjunctive surgery Ann Intern Med 1988 108 4 513 8 2450500 10.7326/0003-4819-108-4-513

[B89] Murphy BR Breeden ES Donohue JP Messemer J Walsh W Roth BJ Einhorn LH Surgical salvage of chemorefractory germ cell tumors J Clin Oncol 1993 11 2 324 9 8381163 10.1200/JCO.1993.11.2.324

[B90] De Santis M Becherer A Bokemeyer C Stoiber F Oechsle K Sellner F Lang A Kletter K Dohmen BM Dittrich C Pont J 2-18fluoro-deoxy-D-glucose positron emission tomography is a reliable predictor for viable tumor in postchemotherapy seminoma: an update of the prospective multicentric SEMPET trial J Clin Oncol 2004 22 6 1034 9 10.1200/JCO.2004.07.188 15020605

[B91] George DW Foster RS Hromas RA Robertson KA Vance GH Ulbright TM Gobbett TA Heiber DJ Heerema NA Ramsey HC Thurston VC Jung SH Shen J Finch DE Kelley MR Einhorn LH Update on late relapse of germ cell tumor: a clinical and molecular analysis J Clin Oncol 2003 21 1 113 22 10.1200/JCO.2003.03.019 12506179

[B92] Djeffal C Demailly M Tillou X Saint F Petit J Place of serum HCG assay in the follow-up of non-HCG-secreting testicular seminomas Prog Urol 2008 18 10 654 6 10.1016/j.purol.2008.04.024 18971108

[B93] Hori K Uematsu K Yasoshima H Yamada A Sakurai K Ohya M Testicular seminoma with human chorionic gonadotropin production Pathol Int 1997 47 9 592 9 10.1111/j.1440-1827.1997.tb04547.x 9311009

[B94] Mirimanoff RO Sinzig M Krüger M Miralbell R Thöni A Ries G Bosset JF Bernier J Bolla M Nguyen TD Prognosis of human chorionic gonadotropin-producing seminoma treated by postoperative radiotherapy Int J Radiat Oncol Biol Phys 1993 27 1 17 23 10.1016/0360-3016(93)90416-S 8365938

[B95] Rüther U Rothe B Grunert K Bader H Sessler R Nunnensiek C Rassweiler J Lüthgens M Eisenberger F Jipp P Role of human chorionic gonadotropin in patients with pure seminoma Eur Urol 1994 26 2 129 33 7525293 10.1159/000475361

[B96] Neill M Warde P Fleshner N Management of low-stage testicular seminoma Urol Clin North Am 2007 34 2 127 36 10.1016/j.ucl.2007.02.009 17484918

[B97] Bruns F Raub M Schaefer U Micke O No predictive value of beta-hCG in patients with stage I seminoma-results of a long-term follow-up study after adjuvant radiotherapy Anticancer Res 2005 25 3A 1543 6 16033057

[B98] Weissbach L Bussar-Maatz R Löhrs U Schubert GE Mann K Hartmann M Dieckmann KP Fassbinder J Prognostic factors in seminomas with special respect to HCG: results of a prospective multicenter study.Seminoma Study Group Eur Urol 1999 36 6 601 8 10.1159/000020055 10559615

[B99] Belkacémi Y Zouhair A Ozsahin M Azria D Mirimanoff RO Réseau des Cancers Rares (Rare Cancer Network) Prognostic factors and management of rare cancers Cancer Radiother 2006 10 317 322 10.1016/j.canrad.2006.07.009 16952474

